# Early warning signal reliability varies with COVID-19 waves

**DOI:** 10.1098/rsbl.2021.0487

**Published:** 2021-12-08

**Authors:** Duncan A. O'Brien, Christopher F. Clements

**Affiliations:** School of Biological Sciences, University of Bristol, Bristol BS8 1TQ, UK

**Keywords:** bifurcation, coronavirus, critical transition, forecasting, monitoring, pandemic

## Abstract

Early warning signals (EWSs) aim to predict changes in complex systems from phenomenological signals in time series data. These signals have recently been shown to precede the emergence of disease outbreaks, offering hope that policymakers can make predictive rather than reactive management decisions. Here, using a novel, sequential analysis in combination with daily COVID-19 case data across 24 countries, we suggest that composite EWSs consisting of variance, autocorrelation and skewness can predict nonlinear case increases, but that the predictive ability of these tools varies between waves based upon the degree of critical slowing down present. Our work suggests that in highly monitored disease time series such as COVID-19, EWSs offer the opportunity for policymakers to improve the accuracy of urgent intervention decisions but best characterize hypothesized critical transitions.

## 1. Introduction

As with many natural systems, the emergence of infectious disease is often sudden and nonlinear, making it difficult for policymakers to identify and appropriately manage threats [1,2]. Balancing the health risks posed by novel diseases and the possible economic impact of imposing mitigation strategies is therefore a complicated process ultimately dependent on the timing of strategy implementation [3].

The severe acute respiratory syndrome-coronavirus 2 (SARS-CoV-2), which causes the coronavirus disease 2019 (COVID-19), exemplifies this challenge. There has been widespread criticism, in response to the harm the pandemic has inflicted, of the speed and severity of the national strategies to the virus threat [4,5]. To date, governments have imposed a spectrum of clinical (e.g. intensive care unit construction), non-pharmaceutical (e.g. large-scale lockdowns) and, most recently, vaccination-based strategies, with the timing of implementation dramatically influencing the case curve between legislative regions [6]. The optimum moment of action is unclear, however, with suggestions that, due to periods of cryptic transmission [7,8], strong non-pharmaceutical interventions (NPIs) two weeks earlier than performed may have halved cumulative deaths [9,10]. Identifying this cryptic window would therefore improve strategy decisions involving possible NPIs based upon the relevant COVID-19 situation.

Unfortunately, the causes of disease emergence and re-emergence often appear idiosyncratic [11], requiring the use of context-specific models [12,13] or risk assessments limited to initial emergence only [14]. These methods are powerful tools and have become keystones during the COVID-19 pandemic response, but are restricted by data availability [15,16] and potential for the lack of transparency [17]. Due to these difficulties, there have been suggestions to consider disease emergence as critical transitions [18] where a forcing pressure, such as host movement or pathogen evolution, drives the system towards a threshold. If considered in this manner, then a suite of alternative methods based upon the concept of critical slowing down (CSD) becomes applicable in the identification of transitions in disease and, potentially, COVID-19 dynamics.

CSD represents the compromised ability of a system to recover from perturbation as it approaches a threshold at which a small perturbation in state triggers a positive feedback loop and system shift [19]. From this phenomenon, various mechanism-independent and summary statistic indicators have been identified across a range of simulated [20,21] and empirical [22,23] studies. In disease systems, CSD was established as tracking a transcritical transition in the effective reproductive number, *R*_eff_, [24] or number of secondary infections an infectious individual causes. Below one, secondary infection is unlikely whereas above one, transmission is common and an outbreak occurs. The period where *R*_eff_ increases towards one is represented by a region of ‘stuttering’ transmission [25] during which CSD also increases [24,26]. ‘Early warning signal’ (EWS) summary statistics based upon CSD will therefore precede the rapid increase in cases at the beginning of an outbreak [27].

EWSs have been shown to predict the emergence of diseases empirically and in simulations [24,28,29]. Variance and autocorrelation increased prior to malaria resurgence in Kenya [28] and before the initial emergence of COVID-19 in seven of the nine countries assessed [30]. In the latter, the exponential increase in cases resulted from the inherently high *R*_eff_ of the SARS-CoV-2 pathogen (greater than 1) [31] rather than a transcritical transition. CSD is therefore not conceptually anticipated to be present [32]. However, wider research has suggested that CSD-based signals may be identifiable not only before a critical transition, but also prior to strong nonlinearity [20,33]; even in the absence of a defined bifurcation, EWSs may detect rapid case increases. This supposition has since been supported by simulated work showing EWSs increase during early stages of exponential and logistic change [34], implying a circumstance exploitable during disease monitoring to identify both exponential growth and transcritical transitions. For the successive COVID-19 re-emergences, however, NPIs restricting *R*_eff_ [35] have theoretically introduced a period of stuttering transmission to cause CSD, and EWSs may act as expected. Thus, COVID-19 represents a unique opportunity to test the efficacy of EWSs in multiple sequential outbreaks by assessing the wave-like dynamics expressed in most countries [36].

Here, we test whether EWSs can predict sequential increases in COVID-19 daily cases, developing a novel methodology to detect and subset data into successive waves of infection. Using the suggested framework, we show evidence that EWSs can be identified prior to nonlinear COVID-19 case increases with the second wave best pre-empted, matching the theoretical predictions. Our results provide suggestions on how to use EWSs in a management scenario, where decisions must be made as data are collected, rather than post hoc.

## 2. Methods

### (a) Data availability

Daily COVID-19 case data were collected from the World Health Organization's dashboard (https://covid19.who.int/info/), spanning 30 January 2020 to 9 June 2021. We analysed positive, daily COVID-19 cases rather than cumulative cases as performed by other studies [30], thus allowing us to attempt the prediction of sequential COVID-19 outbreaks. Case data can be inaccurate from incomplete testing and cryptic cases [35], but we wanted to explore the viability of EWSs using the most universally collected and interpreted data type. The data were consequently analysed in its raw form with no detrending performed.

### (b) COVID-19 wave identification

To assess the increase and decline of COVID-19 cases and define nonlinear regions that can be considered ‘waves’, generalized additive models (GAMs) were iteratively fitted to daily cases in an ‘add-one-in’ fashion, using the R [37] package ‘mgcv’ [38] (see electronic supplementary material 1 for further details. The entire workflow is visualized in the electronic supplementary material 1, figure S1). The time series' inflection points, defined by the significant differing of the GAM smooth's first derivatives from zero, as assessed by 95% pointwise confidence intervals [39], were used to identify nonlinear regions of case increase/decrease and wave onset/subsidence.

### (c) Early warning signal calculation

The presence of EWSs was then calculated using the framework suggested by [40] and developed in [23,41]. Briefly, this approach differs from rolling window EWS methodologies [32,42] by assessing change in an expanding window via a composite metric consisting of multiple indicators. Here, we focussed on the two most used EWS indicators, variance (represented by the standard deviation, s.d.) and autocorrelation at first lag (acf) [43], as well as skewness (skew), following the initial authors [40]. Each indicator was normalized by subtracting its expanding mean from its calculated value at time *t* before dividing by its expanding standard deviation [23]. A composite metric was then constructed by summing all individual indicator values calculated per *t*. An EWS was considered present when the composite metric exceeded its expanding mean by 2*σ* [40]. The 2*σ* threshold was chosen based upon its equivalency to a 95% confidence interval and repeatedly favourable performance compared to other threshold levels [23,41].

As the expanding mean is the basis of assessment, a previous wave will often mask the appearance of second (electronic supplementary material 2, figure S2). Consequently, once a wave subsided, as assessed by GAM first derivatives, the data were cut and the EWS assessment-GAM fitting restarted, truncating the time series from the point of wave end. This resulted in a series of EWS and GAM assessments, each for a specific wave and independent from previous waves. Additionally, the expanding window approach is initially susceptible to false-positive signals due to the short time series length and high variability when few data points are supplied to the algorithm. To mitigate this, a seven-time step burn-in period was introduced to ‘train’ the signals.

Evidence suggests that a persistent signal for two time steps is sufficient to reduce the frequency of false positives [44] and so a ‘warning’ was acknowledged when signals were detected for two consecutive time steps. From the calculated indicators, we present both the individual indicator strengths over time as well as the difference between the time-of-first-detection and the estimated onset of nonlinearity in the UK and abroad. We also identify the superior indicator or combination of indicators for specifically pre-empting COVID-19 case increase.

## 3. Results

**Figure 1.**
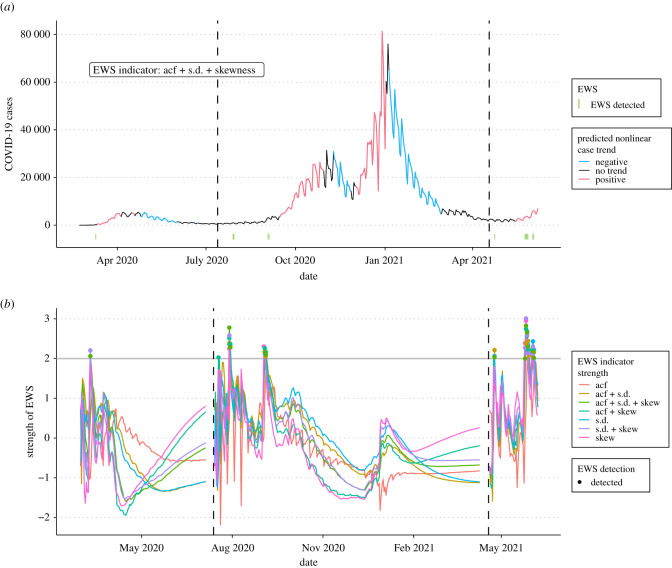
Time series of (*a*) the daily UK COVD-19 cases and sequential GAMs' predicted trends, where green bars represent detected EWSs from the triple composite indicator ‘acf + s.d. + skew’. (*b*) Individual EWS indicator strengths, where coloured dots indicate time points exceeding the 2*σ* threshold (horizontal grey line). Dashed, vertical lines indicate the initialization of GAM and EWS reassessment.

Using the UK as a case study, GAMs predicted three significantly increasing regions in daily COVID-19 cases. These regions correspond to the onset of Waves 1, 2 and, tentatively, 3 beginning on 9 March 2020, 15 September 2020 and 18 May 2021, respectively. From this prediction, two restarts of the EWS-GAM analysis were performed on 7 July 2020 and 12 April 2021 following the subsidence of each wave. Only ‘s.d. + skew’ and the composite metric exceeded the 2*σ* threshold at least once during each UK wave (figure 1). Under our definition of a ‘warning’, Wave 1 was un-detected, whereas Wave 2 was pre-empted by 48 days and indicators lagged Wave 3 by 10 days.

**Figure 2.**
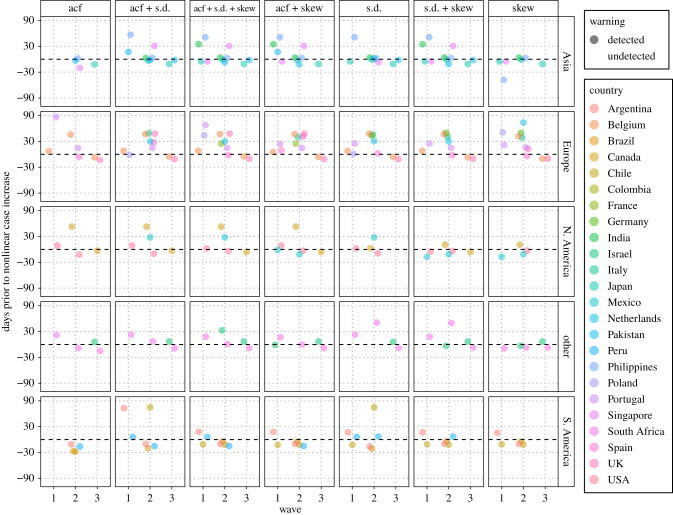
The timeliness spread of individual EWS indicators across a range of countries, grouped by continent (‘other’ consists of Africa and the Middle East). Points represent the number of days that an EWS was detected prior to estimate nonlinear case increases. Failed detections are not plotted. EWS indicators include autocorrelation (acf), variance (s.d.) and skewness (skew).

Similar results were observed in 24 other countries, with GAMs predicting at least two significantly nonlinear increases in each country's daily COVID-19 cases (excluding Portugal). Nine countries also exhibited a third increase (electronic supplementary material 2, figure S3) and Japan alone exhibited a fourth increase. When warnings were averaged between countries, each sequential wave was more weakly pre-empted, although the first wave had the highest false-negative rate of all waves across all indicators (electronic supplementary material 2, table S1, *μ* ± s.e.: Wave 1 = 0.62 ± 0.02, Wave 2 = 0.27 ± 0.01, Wave 3 = 0.22 ± 0.01). The composite metric was the most robust indicator of both emergence and re-emergence (electronic supplementary material 2, table S1; figure 2) with a false negative rate of approximately 0.28 compared to approximately 0.37 for variance, approximately 0.43 for skewness and approximately 0.53 for autocorrelation. The composite's mean detection time did decrease from a 14.6 day pre-emption of COVID-19 emergence to 13.2 days for first re-emergence (Wave 2) and 5.0 day lag for second re-emergence (electronic supplementary material 2, table S1).

European countries displayed the earliest mean pre-emption of the first wave when assessed using the composite metric (figure 2; +26.0 ± 2.8 days), but with a high false-negative rate (0.78) compared to Asia (0.2) which displayed the second earliest mean pre-emption (+19.0 ± 5.7 days). European countries also pre-empted the second wave earliest (+27.2 ± 2.1 days, 0.33 false negative) although no region pre-empted the third wave; other (Africa and the Middle East) exhibited the shortest lag (−0.5 ± 5.3 days, 0.00 false negative).

## 4. Discussion

These results show that EWSs based on the theory of CSD are sufficient to predict the emergence and re-emergence of COVID-19 in multiple countries, but that their ability is context dependent. Indicator ability is improved when time series are subset into individual waves unbiasedly estimated using an iterative GAM approach (figure 1; electronic supplementary material 2, figure S2) but, when EWSs are detected, they only appear for short periods before disappearing. This weakens the practicality of EWSs as a predictive tool for policymakers, as only a few warnings may appear prior to the nonlinear increase. Nevertheless, the observed degree of pre-emption supports EWSs use for characterizing both exponential growth at emergence and later periods of ‘stuttering’ transmission.

Time series variance consistently predicted nonlinear case increase, implying that the indicator is the most informative for characterizing disease outbreaks. This is congruent with previous disease [28,45] and ecological [23,43] findings. The relative weakness of autocorrelation was unexpected considering those studies also identified autocorrelation as a reliable indicator, yet the improvement of the triple composite EWS regardless highlights the benefit of the composite metric approach developed by [40] over individual indicators.

Although this study provides evidence for the partial success of EWS indicators during COVID-19 re-/emergence, their application is often hampered by the quality of data available [46]. Epidemiological data can be particularly problematic due to the reporting style of practitioners. Clinical testing effort changes over time [47] and case data can be aggregated into weekly or monthly counts with the exact date of infection of an individual unknown and many cases being cryptic due to a lack of symptoms [35]. While EWSs based upon disease incidence often display unique behaviour compared to the same indicators based upon disease prevalence [29], this study suggests EWSs can detect changes in daily data via an expanding window and is supported by other studies implementing the alternative rolling window approach [29,48]. COVID-19 case data from Europe does provide a best-case example for EWS assessment, due to the frequency of testing and defined periods of stationarity enforced by NPIs resulting in improved pre-emption compared to other regions (figure 2), but as many governments alter their approach towards future pandemics [49], the data quality may become universal.

The variable pre-emption between waves reported here is particularly important as, statistically, the lag between transition and disease emergence is not fully understood [27]. In this study, it is likely that second re-emergence is not pre-empted due to the shrinking stationary period between it and the previous wave, which in some circumstances may be only one to two weeks in length (e.g. Argentina, Colombia, India). This supports Dablander and colleagues' suggestion [32] that some ‘settling down’ of infection rates is required for EWSs to be persistent and pre-emptive, further weakening their generic usage. Similarly, first re-emergence displaying lowest false-negative EWS rates is consistent with critical theory; wave 2 is anticipated to be the only transcritical transition observed in the time series and so is the only wave thought to display CSD. We therefore suggest that while composite EWSs are not universally detectable prior to COVID-19 waves, they may supplement routine trend analysis in the current monitoring toolbox, alongside other possible early indicators (social media activity—[50], viral shedding in sewerage—[51]), as indicative of persistent nonlinear case growth rather than solely critical transitions.

In conclusion, we suggest that composite EWS indicators may provide useful predictive tools during monitoring, particularly where cases are maintained at low variability for extended periods. When waves rapidly follow one another, CSD-based tools can indicate strong nonlinearity, but these signals are most reliable when *R*_eff_ is maintained sufficiently low that re-emergence results from a transcritical transition. These results support wider notions that EWSs are best suited as a ‘preliminary analysis' indicative of a system at risk and requiring intervention consideration. Although we advocate the use of sequential EWS assessment if used for disease monitoring, it is harder to apply this approach in multi-dimensional systems where a stationary period is likely unidentifiable. Ecosystems, for example, are constantly fluctuating in response to intrinsic or extrinsic drivers [52], with it unclear the minimum length of time series to definitively identify the system's trend [53]. Nonetheless, if periods of stationarity can be identified, we believe sequential assessment is necessary for EWS usage to prevent bias from historic transitions.

## Data accessibility

All data are available from the World Health Organization's dashboard (https://covid19.who.int/info/). Code for the generation of all results and figures are available at https://doi.org/10.5281/zenodo.5556901 [54]. Additional supporting information and data are provided in the electronic supplementary material [55].

## References

[1] Howard CR, Fletcher NF. 2012 Emerging virus diseases: can we ever expect the unexpected? Emerging Microbes Infections **1**, e46. (10.1038/emi.2012.47) 26038413 PMC3630908

[2] Morens DM, Folkers GK, Fauci AS. 2004 The challenge of emerging and re-emerging infectious diseases. Nature **430**, 242-249. (10.1038/nature02759) 15241422 PMC7094993

[3] Anderson RM, Heesterbeek H, Klinkenberg D, Hollingsworth TD. 2020 How will country-based mitigation measures influence the course of the COVID-19 epidemic? Lancet **395**, 931-934. (10.1016/S0140-6736(20)30567-5) 32164834 PMC7158572

[4] Cairney P. 2021 The UK government's COVID-19 policy: assessing evidence-informed policy analysis in real time. Br. Politics **16**, 90-116. (10.1057/s41293-020-00150-8) PMC7603640 38624495

[5] Devine D, Gaskell J, Jennings W, Stoker G. 2020 Trust and the coronavirus pandemic: what are the consequences of and for trust? An early review of the literature. Political Stud. Rev. **19**, 274-285. (10.1177/1478929920948684) PMC7424609 35082554

[6] Prem K et al. 2020 The effect of control strategies to reduce social mixing on outcomes of the COVID-19 epidemic in Wuhan, China: a modelling study. Lancet Public Health **5**, e261-e270. (10.1016/S2468-2667(20)30073-6) 32220655 PMC7158905

[7] Bedford T et al. 2020 Cryptic transmission of SARS-CoV-2 in Washington state. Science **370**, 571-575. (10.1126/science.abc0523) 32913002 PMC7810035

[8] Li R, Pei S, Chen B, Song Y, Zhang T, Yang W, Shaman J. 2020 Substantial undocumented infection facilitates the rapid dissemination of novel coronavirus (SARS-CoV-2). Science **368**, 489-493. (10.1126/science.abb3221) 32179701 PMC7164387

[9] Ragonnet-Cronin M et al. 2021 Genetic evidence for the association between COVID-19 epidemic severity and timing of non-pharmaceutical interventions. Nat. Commun. **12**, 2188. (10.1038/s41467-021-22366-y) 33846321 PMC8041850

[10] Hale T, Hale AJ, Kira B, Petherick A, Phillips T, Sridhar D, Thompson RN, Webster S, Angrist N. 2020 Global assessment of the relationship between government response measures and COVID-19 deaths. *medRxiv*, 2020.07.04.20145334. (10.1101/2020.07.04.20145334) PMC8270409 34242239

[11] Jones KE, Patel NG, Levy MA, Storeygard A, Balk D, Gittleman JL, Daszak P. 2008 Global trends in emerging infectious diseases. Nature **451**, 990-993. (10.1038/nature06536) 18288193 PMC5960580

[12] Kim Y, Lee S, Chu C, Choe S, Hong S, Shin Y. 2016 The characteristics of Middle Eastern respiratory syndrome coronavirus transmission dynamics in South Korea. Osong Public Health Res. Perspect. **7**, 49-55. (10.1016/j.phrp.2016.01.001) 26981343 PMC4776270

[13] Bertozzi AL, Franco E, Mohler G, Short MB, Sledge D. 2020 The challenges of modeling and forecasting the spread of COVID-19. Proc. Natl Acad. Sci. USA **117**, 16 732-16 738. (10.1073/pnas.2006520117) PMC7382213 32616574

[14] Thompson RN, Gilligan CA, Cunniffe NJ. 2020 Will an outbreak exceed available resources for control? Estimating the risk from invading pathogens using practical definitions of a severe epidemic. J. R. Soc. Interface **17**, 20200690. (10.1098/rsif.2020.0690) 33171074 PMC7729054

[15] Holmdahl I, Buckee C. 2020 Wrong but useful — what Covid-19 epidemiologic models can and cannot tell us. New Engl. J. Med. **383**, 303-305. (10.1056/NEJMp2016822) 32412711

[16] Wood SN, Wit EC, Fasiolo M, Green PJ. 2021 COVID-19 and the difficulty of inferring epidemiological parameters from clinical data. Lancet Infect. Dis. **21**, 27-28. (10.1016/S1473-3099(20)30437-0) 32473661 PMC7255708

[17] Jalali MS, DiGennaro C, Sridhar D. 2020 Transparency assessment of COVID-19 models. Lancet Glob. Health **8**, e1459-e1460. (10.1016/S2214-109X(20)30447-2) 33125915 PMC7833180

[18] Drake JM et al. 2019 The statistics of epidemic transitions. PLoS Comput. Biol. **15**, e1006917. (10.1371/journal.pcbi.1006917) 31067217 PMC6505855

[19] Scheffer M et al. 2009 Early-warning signals for critical transitions. Nature **461**, 53-59. (10.1038/nature08227) 19727193

[20] Kéfi S, Dakos V, Scheffer M, Van Nes EH, Rietkerk M. 2013 Early warning signals also precede non-catastrophic transitions. Oikos **122**, 641-648. (10.1111/j.1600-0706.2012.20838.x)

[21] O'Regan SM, Drake JM. 2013 Theory of early warning signals of disease emergence and leading indicators of elimination. Theoret. Ecol. **6**, 333-357. (10.1007/s12080-013-0185-5) 32218877 PMC7090900

[22] Carpenter SR et al. 2011 Early warnings of regime shifts: a whole-ecosystem experiment. Science **332**, 1079. (10.1126/science.1203672) 21527677

[23] Clements CF, Ozgul A. 2016 Including trait-based early warning signals helps predict population collapse. Nat. Commun. **7**, 10984. (10.1038/ncomms10984) 27009968 PMC4820807

[24] Brett T, Ajelli M, Liu QH, Krauland MG, Grefenstette JJ, van Panhuis WG, Vespignani A, Drake JM, Rohani P. 2020 Detecting critical slowing down in high-dimensional epidemiological systems. PLoS Comput. Biol. **16**, e1007679. (10.1371/journal.pcbi.1007679) 32150536 PMC7082051

[25] Blumberg S, Lloyd-Smith JO. 2013 Inference of R0 and transmission heterogeneity from the size distribution of stuttering chains. PLoS Comput. Biol. **9**, e1002993. (10.1371/journal.pcbi.1002993) 23658504 PMC3642075

[26] Miller PB, O'Dea EB, Rohani P, Drake JM. 2017 Forecasting infectious disease emergence subject to seasonal forcing. Theor. Biol. Med. Model. **14**, 17. (10.1186/s12976-017-0063-8) 28874167 PMC5586031

[27] Dibble CJ, O'Dea EB, Park AW, Drake JM. 2016 Waiting time to infectious disease emergence. J. R. Soc. Interface **13**, 20160540. (10.1098/rsif.2016.0540) 27798277 PMC5095216

[28] Harris MJ, Hay SI, Drake JM. 2020 Early warning signals of malaria resurgence in Kericho, Kenya. Biol. Lett. **16**, 20190713. (10.1098/rsbl.2019.0713) 32183637 PMC7115183

[29] Southall E, Tildesley MJ, Dyson L. 2020 Prospects for detecting early warning signals in discrete event sequence data: application to epidemiological incidence data. PLoS Comput. Biol. **16**, e1007836. (10.1371/journal.pcbi.1007836) 32960900 PMC7531856

[30] Kaur T, Sarkar S, Chowdhury S, Sinha SK, Jolly MK, Dutta PS. 2020 Anticipating the novel coronavirus disease (COVID-19) pandemic. Front. Public Health **8**, 521. (10.3389/fpubh.2020.569669) PMC7494973 33014985

[31] Katul GG, Mrad A, Bonetti S, Manoli G, Parolari AJ. 2020 Global convergence of COVID-19 basic reproduction number and estimation from early-time SIR dynamics. PLoS ONE **15**, e0239800. (10.1371/journal.pone.0239800) 32970786 PMC7514051

[32] Dablander F, Heesterbeek H, Borsboom D, Drake JM. 2021 Overlapping time scales obscure early warning signals of the second COVID-19 wave. *medRxiv*, 2021.07.27.21261226. (10.1101/2021.07.27.21261226) PMC8825995 35135355

[33] Dutta PS, Sharma Y, Abbott KC. 2018 Robustness of early warning signals for catastrophic and non-catastrophic transitions. Oikos **127**, 1251-1263. (10.1111/oik.05172)

[34] Jäger G, Füllsack M. 2019 Systematically false positives in early warning signal analysis. PLoS ONE **14**, e0211072. (10.1371/journal.pone.0211072) 30726240 PMC6364907

[35] Flaxman S et al. 2020 Estimating the effects of non-pharmaceutical interventions on COVID-19 in Europe. Nature **584**, 257-261. (10.1038/s41586-020-2405-7) 32512579

[36] Pedro SA, Ndjomatchoua FT, Jentsch P, Tchuenche JM, Anand M, Bauch CT. 2020 Conditions for a second wave of COVID-19 due to interactions between disease dynamics and social processes. Front. Phys. **8**, 428. (10.3389/fphy.2020.574514)

[37] R Core Team. 2021 R: a language and environment for statistical computing. Vienna, Austria: R Foundation for Statistical Computing. See https://www.R-project.org/.

[38] Wood SN. 2011 Fast stable restricted maximum likelihood and marginal likelihood estimation of semiparametric generalized linear models. J. R. Stat. Soc. **73**, 3-36. (10.1111/j.1467-9868.2010.00749.x)

[39] Simpson GL. 2021 gratia: graceful ‘ggplot’-based graphics and other functions for GAMs fitted using ‘mgcv’.

[40] Drake JM, Griffen BD. 2010 Early warning signals of extinction in deteriorating environments. Nature **467**, 456-459. (10.1038/nature09389) 20827269

[41] Clements CF, Blanchard JL, Nash KL, Hindell MA, Ozgul A. 2017 Body size shifts and early warning signals precede the historic collapse of whale stocks. Nat. Ecol. Evol. **1**, 188. (10.1038/s41559-017-0188) 28812591

[42] Dakos V et al. 2012 Methods for detecting early warnings of critical transitions in time series illustrated using simulated ecological data. PLoS ONE **7**, e41010. (10.1371/journal.pone.0041010) 22815897 PMC3398887

[43] Dakos V, van Nes EH, D'Odorico P, Scheffer M. 2012 Robustness of variance and autocorrelation as indicators of critical slowing down. Ecology **93**, 264-271. (10.1890/11-0889.1) 22624308

[44] Clements CF, McCarthy MA, Blanchard JL. 2019 Early warning signals of recovery in complex systems. Nat. Commun. **10**, 1681. (10.1038/s41467-019-09684-y) 30975997 PMC6459826

[45] O'Regan SM, Lillie JW, Drake JM. 2016 Leading indicators of mosquito-borne disease elimination. Theoret. Ecol. **9**, 269-286. (10.1007/s12080-015-0285-5) 27512522 PMC4960289

[46] Clements CF, Drake JM, Griffiths JI, Ozgul A. 2015 Factors influencing the detectability of early warning signals of population collapse. Am. Nat. **186**, 50-58. (10.1086/681573) 26098338

[47] Mercer TR, Salit M. 2021 Testing at scale during the COVID-19 pandemic. Nat. Rev. Genet. **22**, 415-426. (10.1038/s41576-021-00360-w) 33948037 PMC8094986

[48] Brett TS, O'Dea EB, Marty É, Miller PB, Park AW, Drake JM, Rohani P. 2018 Anticipating epidemic transitions with imperfect data. PLoS Comput. Biol. **14**, e1006204. (10.1371/journal.pcbi.1006204) 29883444 PMC6010299

[49] Coccia M. 2021 The relation between length of lockdown, numbers of infected people and deaths of Covid-19, and economic growth of countries: lessons learned to cope with future pandemics similar to Covid-19 and to constrain the deterioration of economic system. Sci. Total Environ. **775**, 145801. (10.1016/j.scitotenv.2021.145801)

[50] Yousefinaghani S, Dara R, Mubareka S, Sharif S. 2021 Prediction of COVID-19 waves using social media and Google search: a case study of the US and Canada. Front. Public Health **9**, 359. (10.3389/fpubh.2021.656635) PMC8085269 33937179

[51] Bibby K, Bivins A, Wu Z, North D. 2021 Making waves: plausible lead time for wastewater based epidemiology as an early warning system for COVID-19. Water Res. **202**, 117438. (10.1016/j.watres.2021.117438) 34333296 PMC8274973

[52] Dakos V, Carpenter SR, van Nes EH, Scheffer M. 2015 Resilience indicators: prospects and limitations for early warnings of regime shifts. Phil. Trans. R. Soc. B **370**, 20130263. (10.1098/rstb.2013.0263)

[53] Wauchope HS, Amano T, Sutherland WJ, Johnston A. 2019 When can we trust population trends? A method for quantifying the effects of sampling interval and duration. Methods Ecol. Evol. **10**, 2067-2078. (10.1111/2041-210X.13302)

[54] O'Brien D, Clements C. 2021 Early warning signal reliability varies with COVID-19 waves. Zenodo (10.5281/ZENODO.5556901) PMC8651412 34875183

[55] O'Brien DA, Clements CF. 2021 Early warning signal reliability varies with COVID-19 waves. *Figshare*. 10.1098/rsbl.2021.0487 PMC8651412 34875183

